# Depression and its determinants among adolescents in Jimma town, Southwest Ethiopia

**DOI:** 10.1371/journal.pone.0250927

**Published:** 2021-05-03

**Authors:** Shimelis Girma, Mekonnen Tsehay, Almaz Mamaru, Mubarek Abera

**Affiliations:** 1 Department of Psychiatry, Institute of Health, Jimma University, Jimma, Ethiopia; 2 Department of Psychiatry, Wollo University, Dessie, Wollo, Ethiopia; University of the Witwatersrand, SOUTH AFRICA

## Abstract

**Objectives:**

To determine the prevalence and socio-demographic and parental-related factors of depression among school adolescents in Jimma town, southwest Ethiopia.

**Methods:**

Using a cross-sectional survey, 546 school adolescents were screened for depression using the patient health questionnaire (PHQ-9) from five randomly selected public and private schools. Oslo social support scale, adverse childhood experience tool, and socio-demographic questionnaire were used to gather data on risk factors. Linear regression analysis was used, and unstandardized beta (β) coefficients with 95% confidence intervals (CI) were reported to declare statistical significance.

**Results:**

A total of 546 adolescents participated in the study, with a response rate of 97.3%. The mean (±SD) age of participants was 16.8 ± 1.3 years. The majority (81%) of the adolescents were attending day classes at public schools. The prevalence of depression was found to be 28% using the patient health questionnaire. Based on the PHQ-9 depression severity scale, 18.5% and 8.2% of the adolescent had moderate and moderate to severe depression while 1.3% had severe depression. In the final multivariate linear regression analysis, it was found that sex, rural residence, low social support, being in higher grade level, and adverse childhood experience were found to be independently associated with a higher score of depression.

**Conclusion:**

One in three adolescents was found to have a depressive syndrome. We recommend schools to integrate school mental health service that contains routine screening and intervention services. Moreover, efforts are needed to sensitize and educate the communities on child protection, social support, and safeguarding to effectively tackle the magnitude of adolescent depression.

## Introduction

Adolescence, a developmental period between ages 10–19 years, is a period in which individuals transit from childhood to adulthood. It involves the main transformations in the domain of physical, psychological, social, and cognitive development. This change is associated with a state of role confusion, high-level stress, and emotional instability [1]. Global mental health burden indicated that 10–20% of adolescents were affected by at least one type of mental disorder [2]. In 2018, World Health Organization (WHO) reported that more than 1 million adolescents died from preventable causes [3], and depression is a significant contributor to mortality and morbidity in these age groups [4]. Studies from different parts of the world showed that depression among adolescents is rising and becomes the most common psychiatric disorder among adolescents [5].

Studies regarding adolescent depression are mainly limited to high-income countries. A study conducted in the United States found out that 14% and 12.5% of study participants met the criteria for a mood disorder and depressive disorder, respectively [6]. A study done on young Europeans found out one in ten adolescents showed significant depressive symptoms [7], and a similar rate was reported in Germany [8]. There have been few studies in low-and middle-income countries (LAMICs) on adolescent depression. Studies on adolescent depression in sub-Saharan African countries documented a magnitude of 15.3–37% in Egypt [9, 10], 14.7–72% in Iran [11, 12], 40% in India [13], and 31.5% in Malaysia [14]. A few studies done in Eastern Africa showed a higher prevalence; it was 26.4% in Kenya [15], and 21% in Uganda [16].

Available literature indicated that parental and socio-demographic factors such as age, parents’ occupational status [17], female sex [18], lower parental education and economic status [19], and risky behaviors (substance use) [20] were independently associated with adolescent depression. Evidence from the systematic review has shown that depression at an earlier age is associated with poor illness outcomes, impaired social and academic functioning, and recurrent depressive episodes during adulthood [21]. More than 40% of disability-adjusted life years (DALYs) attributed to mental and substance use disorders [22] and increased mortality due to suicide [23].

Though the magnitude of adolescent depression and its consequence on adult health and wellbeing is high, depression symptoms in adolescents are rarely recognized and managed. The symptoms are considered as either a familiar life experience or naturalist behavioral transition [24]. Diagnosis of adolescent depression is also challenging as the predominant presentation is somatic or physical symptoms [25]. Furthermore, the nature of the presentation is characterized by prominent irritability, mood reactivation, and the fluctuation of symptoms unlike typical depressive presentation in an adult [26].

In Ethiopia studies on adolescent depression are very limited and those available are focused on a vulnerable group of adolescents such as orphans, and adolescents living with human immunodeficiency virus (HIV) [27–29]. This study is the first in its type to address factors like adverse childhood experience (ACE), social support, and substance use with adolescent depression. Therefore, the purpose of this study was to determine the prevalence of depression and identify socio-demographic and parental-related factors among school-going adolescents in Jimma town.

## Methods and materials

### Study design and study setting

A school-based cross-sectional study design was employed from 2, April to 30, May 2018. This study was conducted in Jimma town, Oromia regional state. The region has diverse agro-ecological zones. The community in the region is mainly engaged in rain-fed agriculture and livestock rearing. The poverty headcount ratio in the region was 23.9% [30]. The primary school net enrolment rate (Grades 1–8) in the region is 94.7% and the illiteracy rate (15-17years) is 47.6% [31]. The town is situated in the southwest of Ethiopia and covers a total area of 4,623 hectares and the daily mean temperature of the town ranges from 20°c to 25°c year-round and the average annual rainfall is 1500mm. Coffee farming is the most favored and dominant form of livelihood in the area [32]. The town has six public and eight private high schools (grades 9–12) with a total of 9,383 students registered for the academic year of 2018/2019. More than three-fourths of students, 7,292 were from public and the remaining 2,091 were from private schools.

### Population

All adolescents aged 10–19 years who were attending secondary school education in Jimma town were considered the source population. The study population was 546 randomly selected students from two public and three private secondary schools that were aged 10–19 years.

### Sample size determination and sampling techniques

**Fig 1:**
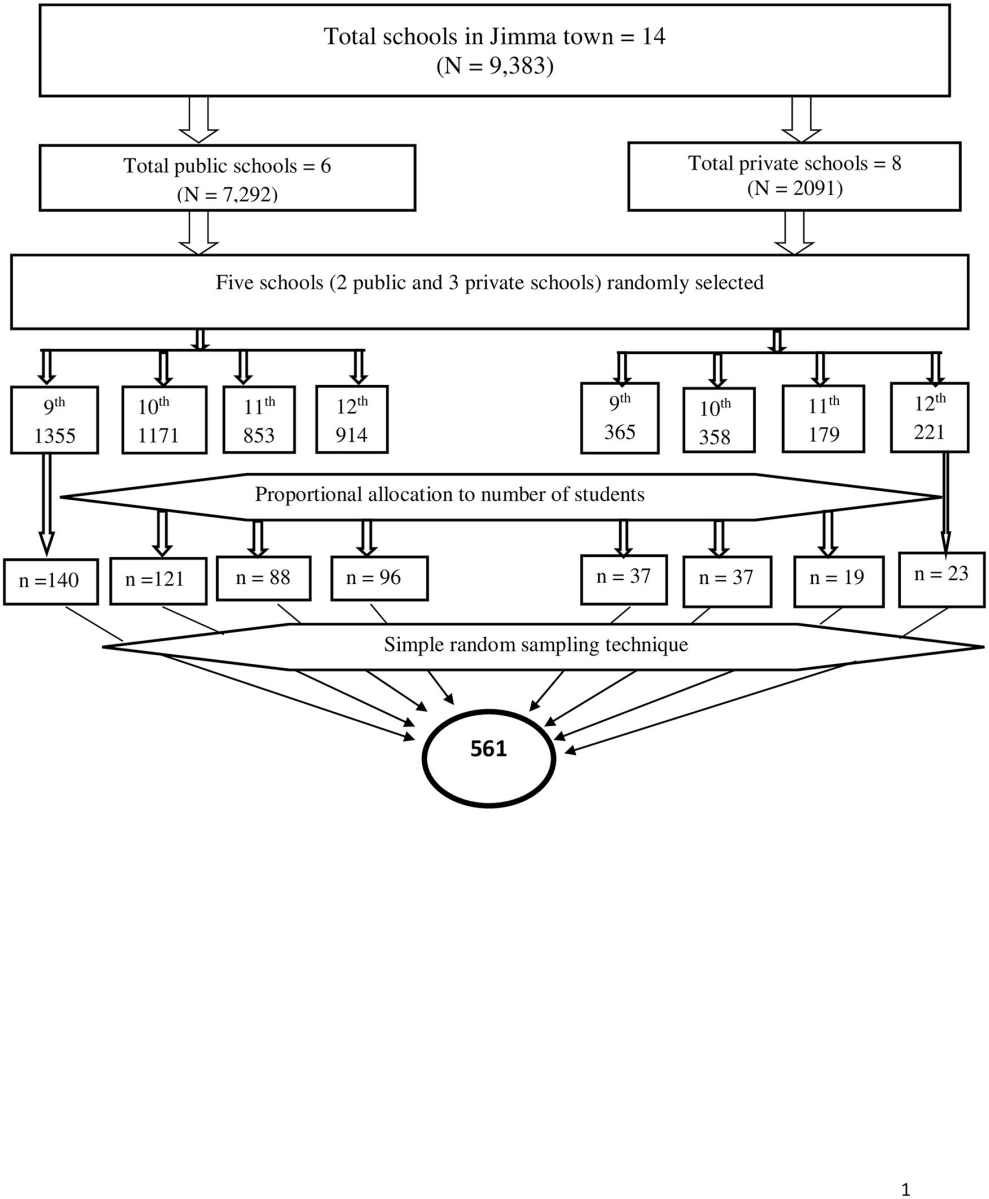
Schematic representation used in the selection of school-going adolescent in Jimma town, Southwest Ethiopia (n = 546).

The sample size was calculated using a single population proportion, taking into account the following assumptions: the prevalence of depression among adolescent students 21.0% [16], a margin of error 5%, 95% confidence interval, 10% non-response rate, and a multi-stage effect of two. Thus, the minimum sample size required for the study was found to be 561. A multi-stage sampling technique using simple random sampling was used in this study. At the first stage, schools were stratified into public and private. The public schools in the town were six in number, and the private schools were eight. Schools are stratified into public and private schools with the assumption that there is a socio-economic difference between study subjects attending public and private schools. Two public schools and three private schools were randomly selected. In the second stage, students from randomly selected schools were stratified into grade levels from grades 9 to 12. Following the proportional allocation of study participants based on class size, students were randomly selected from each grade level using student registers. The likelihood of adolescents attending private and public schools being selected into the study was 0.05% and 0.06%, respectively (Fig 1).

### Data collection procedure and measurements

The data were collected using a structured self-administered questionnaire. The questionnaire included socio-demographic characteristics, substance use, social support, adverse childhood experience, and depressive symptom assessment. Adolescent depression assessed using a patient health questionnaire (PHQ-9A) [33]. PHQ-9A is a depression screening tool that has nine items. The tool was previously translated into several languages, including Afaan Oromo and Amharic, and it was validated in both languages in the Ethiopian adult population and showed a sensitivity of 86% and specificity of 67% [34]. PHQ-9 score >10 had a sensitivity of 89.5%, and specificity of 77.5%. The cut-off score allows consistency in diagnosing depression with the Diagnostic Statistical Manual (DSM-V) criteria for major depression [35]. The tool demonstrated high reliability in this study, with Cronbach’s alpha (α = 0.84). An adverse childhood experience (ACE) questionnaire was used to evaluate adolescents’ childhood experiences. The tool is a self-report instrument that has ten items intended to rate the severity of emotional abuse, neglect, physical abuse, and sexual abuse [36]. Each student also reported to self—rated health questions, on a Likert scale with five points (“poor” = 0, “fair” = 1, “good” = 2, “very good” = 3, “excellent” = 4). Substance use assessed using the World Health Organization Alcohol, Smoking, and Substance Involvement Screening test (WHO ASSIST) version 3.1. It has 8-items and is culturally neutral and useable across a variety of cultures to assess the use of psychoactive substances [37]. The Oslo 3-items social support scale was used to assess social support [38]. An index was made by summarizing the sum of raw scores, and the total score ranged from 3 to 14. The ethical aspect ensured by providing information to the adolescents and information sheet together with the consent form was sent to the family to obtain the consent of the family days before data collection. Classroom teachers facilitate the distribution of study questionnaires for selected students during school hours. The tool was self-administered in a local language.

### Statistical analysis

Data were entered into EpiData version 3.1 and exported to Statistical Package for Social Sciences (SPSS) version 23.0 for analysis. Descriptive statistics, such as frequency, mean, and standard deviations were computed. Linear regression analysis was used to determine independent predictors of depression. Assumptions for linear regression assessed: linearity was checked by a scatter plot, and it depicted no significant outlier; normality of distribution checked by a histogram, and well fitted the normal distribution curve. Furthermore, multicollinearity was checked, and the Pearson correlation coefficient and variance inflation factor (VIF) found to be < 1. Bivariate/simple linear regression analysis done and variables with p-value < 0.25 were considered for multiple linear regression. Multiple linear regression analyses were used to identify factors independently associated with depression. Variables that had a P-value < 0.05 were declared significantly associated with depression. Unstandardized beta (β) coefficients with 95% confidence intervals (CI) were computed to assess the level of association and statistical significance in multiple linear regression analyses.

### Ethical approval and consent to participate

Ethical approval was obtained from the Institutional Review Board (IRB) of Jimma University Institute of Health. The ethical approval letter was submitted to all the schools and permission obtained from the school governing bodies. Following permission from the school directors, students took the parent/guardian information sheet and were told to return after the parent or guardian complete the consent form prepared in the local language (Amharic or Afaan Oromo) days before data collection. A detailed participant information sheet given to each student, and they completed an assent form to indicate a willingness to participate. Only students who completed an assent and parental/guardian consent form were eligible to participate. Adolescents were found to have moderate and severe depression linked to school counselors and Jimma Medical Center psychiatry clinic.

## Result

### Sociodemographic characteristics of the participants

From a total of 561 adolescents invited to participate, 546 adolescents completed the questionnaire. Fifteen participants submitted either incomplete or inconsistent survey questionnaire, and it was not included in the analysis. Thus, the response rate of the study was 97.3%. Four-fifth of study participants 442 (81%) were from public schools. The mean age of adolescents was 16.83 years, standard deviation (SD) ±1.3 years, and ranged from 14 to 19 years. About half (44%) were Muslim. In terms of participants’ parental occupation, 219 (40.1%) of the fathers and 135 (24.7%) mothers were merchants and housewives, respectively. Almost all of the fathers attended formal education, while one-tenth of the mothers had no formal education (Table 1).

**Table 1:** Sociodemographic characteristics of school-going adolescents in Jimma town, Southwest Ethiopia (n = 546).

Variables	Category	Frequency	Percent
Age	14	13	2.4
	15	84	15.4
	16	114	20.9
	17	153	28.0
	18	137	25.1
	19	45	8.2
Sex	Female	329	60.3
	Male	217	39.7
Grade	9	174	31.9
	10	153	28.0
	11	103	18.9
	12	116	21.2
Religion	Muslim	240	44.0
	Orthodox Christian	212	38.8
	Protestant	75	13.7
	Others (catholic and wakefata)	19	3.5
School	Governmental	442	81.0
	Private	104	19.0
Occupation of father	Merchant	219	40.1
	Governmental	163	29.9
	Private	124	22.7
	Laborer	40	7.3
Occupation of mother	Government	147	26.9
	Private	137	25.1
	Housewife	135	24.7
	Merchant	93	17.0
	Laborer	34	6.2
Educational status of the father	No formal education	40	7.3
	1–4	69	12.6
	5–8	124	22.7
	9–12	163	29.9
	certificate and above	150	27.5
Educational status of the mother	No formal education	51	9.3
	1–4	96	17.6
	5–8	159	29.1
	9–12	138	25.3
	certificate and above	102	18.7
Place of residence	Urban	450	82.4
	Rural	96	17.6

### Prevalence of depression and experience of depressive symptoms

**Fig 2:**
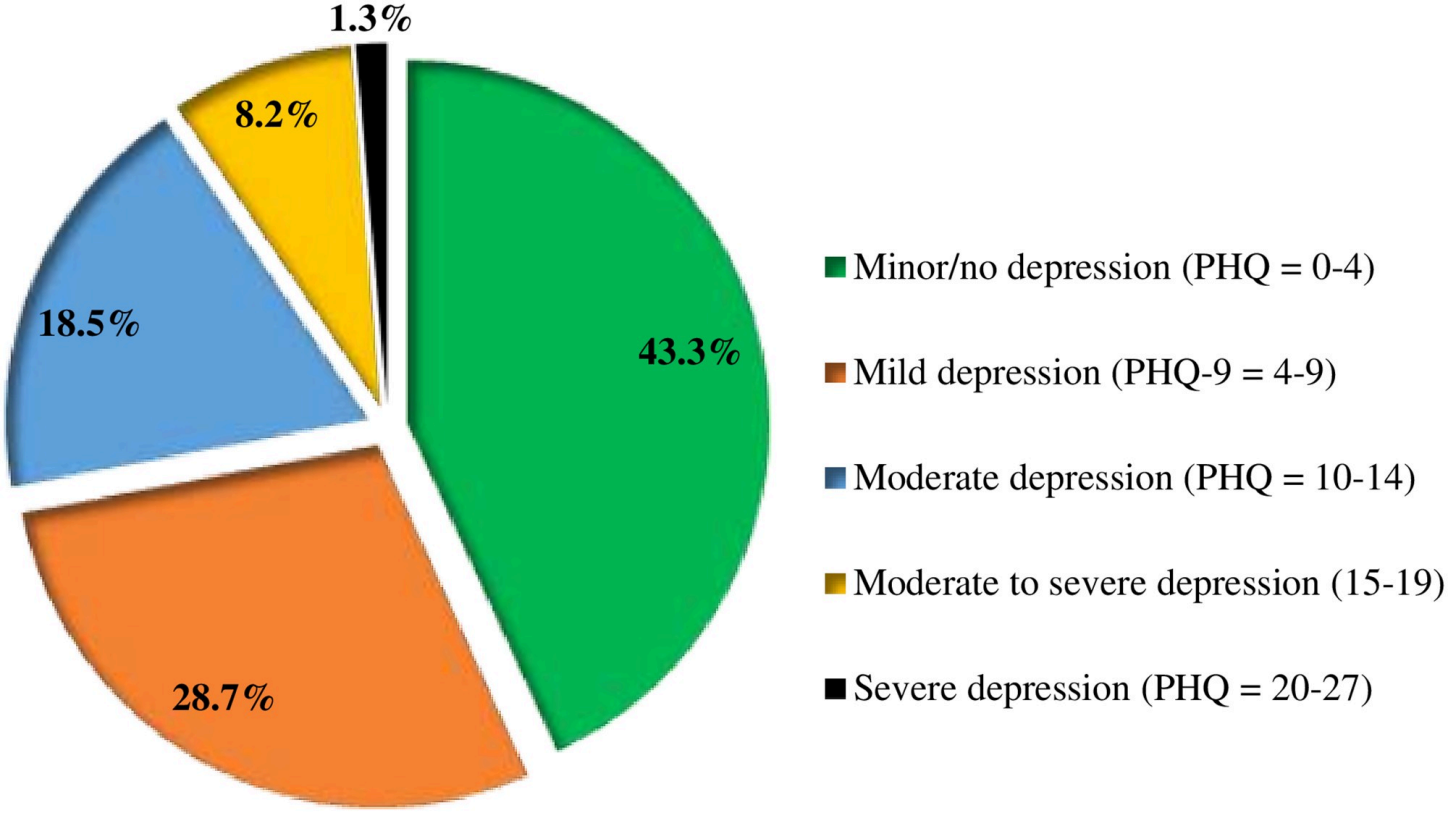
Magnitude and severity of depression among school-going adolescent in Jimma town, Southwest Ethiopia (n = 546).

The prevalence of depression was 28%, 95% CI (24.5, 32.1) using a PHQ-9 cut-off point ≥ 10. Based on the PHQ-9A severity scale, 18.5% and 8.2% of the adolescents had moderate and moderate to severe depression, while, 1.3% had severe depression (Fig 2). Regarding suicidal behavior, 6.4%, 95% CI (4.4, 8.8) had at least one suicidal attempt, while 7%, 95% CI (5.2, 9.8) had a suicidal thought for the last two weeks before the study.

### Factors associated with depression

In the bivariate linear regression analysis, 13 variables such as age, sex, ownership of shools, place of residency, grade level, paternal occupation, maternal occupation, paternal educational status, maternal educational status, co-morbid physical illness, poor self-rated health, adverse childhood experience, and social support were found to be statistically significantly associated with a higher score of adolescent depression. However, in the final adjusted (multiple) linear regression model, only sex, rural residence, social support, grade level, and childhood adverse experience showed statistical significance with a higher score of adolescent depression. While male sex and social support were associated negatively, rural residency, higher school grade, and childhood adverse life experience associated positively with a higher score of adolescent depression. Being male decreased depressive symptoms by 1.09 units (β = -1.09, 95% CI (-0.02, -0.49) compared to female. Residing in rural areas of the town increased the PHQ-9A score by 0.89 (β = 0.89, 95% CI (0.13, 1.65). Moreover, being in grade 12 and having negative childhood life experience increased the PHQ-9A score by 1.14 (β = 1.14, 95% CI (1.29, 1.97) and 0.51 (β = 0.51, 95% CI (0.34, 0.69), respectively. Regarding social support, a unit increase in Oslo-3 social support score decreased PHQ-9A score by 0.27 (β = -0.27, 95% CI (-0.37, -0.17) (Table 2).

**Table 2:** Adjusted multiple linear regression model showing factors independently associated with depression among school-going adolescent in Jimma town, Southwest Ethiopia (n = 546).

Explanatory variables		Simple linear regression		Multiple linear regression	
		Beta coefficients (β)	95% CI for β	Beta coefficients (β)	95% CI for β
Chronological age		0.59	0.20 to 0.99	0.23	-0.02 to 0.49
Sex	Female	Reference group			
	Male	-0.66	-1.35 to 0.03	-1.09	-1.63 to -0.56
Ownership of school	Private school		0		0
	Public school	0.55	-0.32 to 1.4	-0.32	-0.99 to 0.36
Place of residence	Urban	Reference group			
	Rural	1.46	0.57 to 2.34	0.89	0.13 to 1.65
Grade level	Grade 9	Reference group			
	Grade 10	0.63	-.12 to 1.38	0.52	-0.12 to 1.16
	Grade 12	0.95	0.12 to 1.77	1.14	0.29 to 1.97
Occupation of father	Governmental	Reference group			
	Merchant	-1.43	-2.29 to -.56	-0.21	-0.94 to 0.53
	Private	1.12	0.35 to 1.89	-0.04	-0.72 to 0.63
Occupation of mother	Government	Reference group			
	Daily laborer	0.87	-0.53 to 2.27	0.54	-0.63 to 1.72
	Private	0.54	-0.24 to 1.32	0.25	-0.45 to 0.96
	House wife	-0.59	-1.37 to .19	-0.67	-1.34 to -0.01
Educational status father	No formal education	Reference group			
	Primary	0.34	-0.12 to 1.68	0.18	-0.44 to 0.81
	Tertiary	0.71	0.41 to 1.17	0.08	-0.72 to 0.87
Educational status of the mother	No formal education	Reference group			
	Tertiary	-0.59	-1.46 to 0.27	0.05	-0.81 to 0.91
Co-morbid illness		2.86	1.22 to 4.49	0.70	-0.59 to 2.0
Poor self-rated health	Poor	Reference group			
	Fair	0.67	0.29 to 1.04	0.18	-0.13 to 0.49
	Excellent	-0.55	-0.73 to -0.37	-0.16	-0.32 to 0.01
Adverse childhood experience		2.85	2.23 to 3.46	0.51	0.34 to 0.69
Oslo social support		-0.62	-0.73 to -0.49	-0.27	-0.38 to—0.17

## Discussion

The prevalence of depression in the present study is 28%, of whom 18.5%, 8.2%, and 1.3% were found to have moderate, moderate to severe, and severe depression, respectively. In the final multivariate linear regression analysis, female sex, rural residence, low social support, being in higher grade level, and adverse childhood experience found to be independently associated with a higher score of depression.

The overall prevalence of depression in the current study (28%) is in line with previous research findings done in Kenya (26.4%) [5], and Iran (31.30%) [13]. However, the prevalence in the current study is lower than the findings from other studies in India [39], Saudi Arabia [16], and Bangladesh [40], which had reported 57.7%, 46.9%, and 36%, respectively. On the contrary, the prevalence of depression in a study done in the United States of America among school adolescents was 18% [14], whereas, it was 21% and 15.3% in central Uganda [18] and Egypt [9] respectively. Such a degree of difference in the prevalence of depression across the different parts of the world could originate from variations in the depression screening tools used. A study done in central Uganda used the Children Depression Inventory (CDI), and a systematic review of Iranian studies used the Beck Depression Inventory (BDI) and CDI. Furthermore, the difference might be due to the variation in population characteristics. A study done in Bangladesh targeted urban and semi-urban school-going adolescents, and the central Ugandan study included non-boarding, a boarding school for male, and boarding schools for females. Moreover, cultural differences in the different study settings might have also contributed for the variation.

The present study showed variation in the prevalence rate of depression across different socio-demographic variables. Previous studies revealed a disparity in rates of depressive symptoms across sex, where girls experienced depressive symptoms twice more than boys [18, 41]. In line with these findings, our research showed that males experience fewer depressive symptoms (β = -1.09, 95% CI (-1.63, 0.56) than girls. This variation is explained by the biological theory and the differences in the physical makeup of males and females after puberty. Fluctuations in hormonal levels and age-related behavior change put females at risk [42]. Regarding the relationship between age and depression, this study did not find a significant linear relationship between the age of students and depression (β = 0.24, 95% CI (-0.06, 0.06). The finding is in agreement with the study done by Ekundayo *et al*. did not find any variation in the prevalence of depression with age [43].

Studies have persistently reported the association between social support and mental health problems among adolescents [44–46]. Scientific evidence revealed that poor relationships with schoolmates, feeling of insecurity, and adverse life events increase the risk of mental disorders in adolescents [47, 48]. Moreover, a longitudinal study revealed the feeling of connectedness, absence or low levels of conflict, and social environment, which is encouraging to express once the emotion is protective against the occurrence of emotional and behavioral disorders [49, 50]. Another longitudinal study conducted in the USA also revealed a strong effect of life adversities and the negative consequences on adolescent health [51]. In line with this evidence, the current study found out a unit increase in the Oslo-3 social support scale decreases PHQ-9A score by 0.27 (β = -0.27, 95% CI (-0.38, -0.17). This finding is supported by a study done in central Uganda [18], Malaysia [34], and Saudi Arabia [52].

Adverse childhood experiences (abuse, neglect, and household dysfunction) had a significant association with depression. A unit increase in ACE score increased the PHQ-9 score by 0.51 (β = 0.51, 95% CI (0.34, 0.69). Similar findings were reported from the Netherlands and Colombia [53, 54]. Evidence from previous findings showed the existence of a relationship between adolescent socio-emotional and behavioral problems with adverse childhood experiences [55, 56]. This is explained by the psychological insult to the brain that could have impacted the neurobiological and behavioral development during childhood and adolescence. Evidence indicates that exposure to violence and stressors during early life results in a smaller prefrontal cortex and its function, impaired response to stressors, increased inflammatory mediators [57, 58], and gene modification [59]. The major strength observed in this study was the maximum effort exerted to ensure a random selection of study participants. Thus, generalization to adolescent students in the study area is possible. Aside from this, this study is the first in type to address the association of adolescent depression with social support and childhood adverse life experiences in the locality. The cross-sectional nature of the study poses difficulty in evaluating the time temporal relationship between predictors and outcome variables. Tools used for depression tend to overestimate depression at a cut-off point of 10. The subjective assessment of some factors was prone to recall bias, and some of the parental-related factors such as parenting style were not addressed in this study. Furthermore, the sampling was not adjusted for sex.

## Conclusions

One in three adolescents was found to have depression in Jimma town. Female sex, rural residence, low social support, higher grade level, and ACE score were independent predictors of depression. We recommend schools institute routine screening services and implement appropriate interventions to initiate and improve access to school-based mental health services. The local administration needs to exert more efforts to sensitize and educate communities on the importance of social support, child and adolescent protection, and safeguarding.

## Supporting information

S1 Dataset (XLS)
